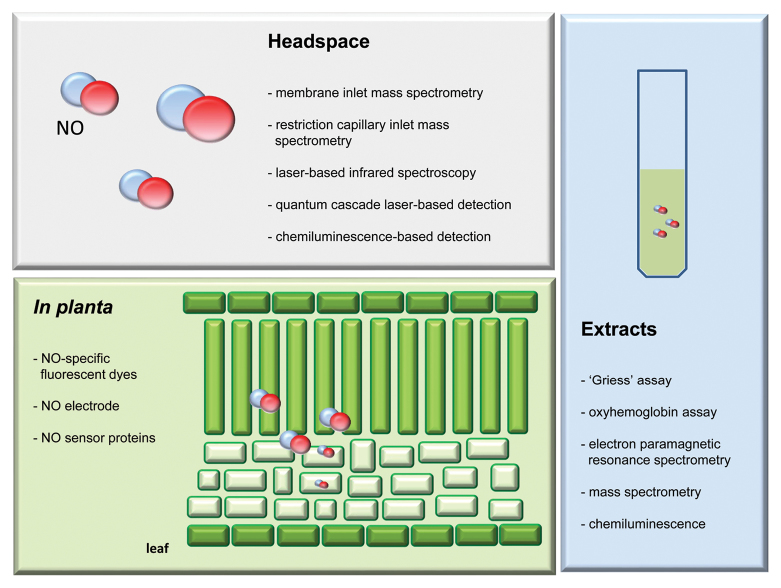# Nitric oxide sensor proteins with revolutionary potential

**DOI:** 10.1093/jxb/ery193

**Published:** 2018-06-27

**Authors:** Christian Lindermayr, Jörg Durner

**Affiliations:** 1 Institute of Biochemical Plant Pathology, Helmholtz Zentrum München – German Research Center for Environmental Health, München/Neuherberg, Germany; 2 Lehrstuhl für Biochemische Pflanzenpathologie, Technische Universität München, Freising, Germany

**Keywords:** Abiotic and biotic stress, electron paramagnetic resonance (EPR) spectroscopy, legume nodules, nitric oxide (NO), nitrosyl–leghemoglobin (Lb^2+^NO), NO detection/production, NO sensor proteins

## Abstract

This article comments on:

Calvo-Begueria L, Rubio MC, Martínez JI, Pérez-Rontomé C, Delgado MJ, Bedmar EJ, Becana M. 2018. Redefining nitric oxide production in legume nodules through complementary insights from electron paramagnetic resonance spectroscopy and specific fluorescent probes. Journal of Experimental Botany **69,** 3703–3714.

**Nitric oxide (NO) is involved in regulation of plant growth and development, as well as the response to biotic and abiotic stressors. However, its instability makes NO methodology a complex and often controversial field. A new method from** Calvo-Begueria *et al.* (2018) **to examine NO production in intact nodules uses electron paramagnetic resonance (EPR) spectroscopy to detect nitrosyl–leghemoglobin (Lb** ^**2+**^ **NO). NO sensor proteins are an optimal tool for NO detection/quantification *in vivo* and have the potential to revolutionize the field of plant NO research.**

Nitric oxide (NO) is an important redox molecule fulfilling a wide variety of signalling functions. These cover growth and development, as well as stress responses, in humans, animals, plants, fungi and bacteria (Besson-Bard *et al.*, 2008; Sudhamsu and Crane, 2009; Murad, 2011; Arasimowicz-Jelonek and Floryszak-Wieczorek, 2016; Byung-Wook *et al.*, 2016; Canovas *et al.*, 2016). In plants, NO is involved in seed germination, root development, gravitropism, iron homeostasis, stomatal closure, flowering, and pollen tube growth (Bellin *et al.*, 2013; Freschi *et al.*, 2013; Simontacchi *et al.*, 2015; Corpas *et al.*, 2017). Moreover, programmed cell death, activation of defence genes and genes involved in UV, heat, drought and salinity stress tolerance require the function of NO (Groß *et al.*, 2013; Kulik *et al.*, 2015; Domingos *et al.*, 2015; Simontacchi *et al.*, 2015; Hu *et al.*, 2017). As a diffusible gas it can be present in all extra- and intracellular spaces, where it easily interacts with the surrounding environment.

NO can be produced by oxidative and reductive pathways (Moreau *et al.*, 2010; Yu *et al.*, 2014) and is sensed within the cell through redox modification of proteins, such as cysteine nitrosation, tyrosine nitration and metal nitrosylation (Astier *et al.*, 2012; Astier and Lindermayr, 2012; Mata-Perez *et al.*, 2016). One of its most important modes of action is protein *S*-nitrosation, the covalent attachment of NO to the thiol group of protein cysteine residues. Tyrosine nitration refers to the addition of a nitro group to susceptible tyrosine residues in the *ortho* position to the hydroxyl group yielding 3-nitrotyrosine. The main nitrating species is peroxynitrite which is produced in a diffusion-controlled reaction between NO and superoxide. In a direct metal nitrosylation reaction, NO (Lewis base) binds to the transition metal (Lewis acid) of metalloproteins yielding a metal–nitrosyl complex.

## Detection difficulties

Understanding the ‘conduct’ of NO in biological systems is important. However, investigation of NO production and NO signalling is challenging because many available methods suffer from a lack of specificity and/or sensitivity, or are just unsuitable for the detection of NO *in vivo*. Additionally, in some cases, the production might be restricted to a few cells, such as guard cells or pollen (Corpas *et al.*, 2004; Prado *et al.*, 2004; Neil *et al.*, 2008). NO is a reactive molecule with a lifetime in the order of seconds to minutes. Moreover, in physiological buffers, it diffuses rapidly with a diffusion coefficient approaching 3300 µm^2^ s^–1^(Malinski *et al.*, 1993; Lancaster, 1997). Thus, any detection method must be very sensitive to be able to chase intraorganismic NO production. In sum, NO research requires a broad spectrum of complementary methods, which together allow an accurate identification of NO and its physiological function.

Sensitive and specific analytical tools for measuring NO *in vivo* are rare. NO-specific fluorescent dyes, electrodes and sensor proteins are the only options for detecting and quantifying NO in living cells/tissues (Arasimowicz *et al.*, 2009; Eroglu *et al.*, 2016). Others, such as the Griess assay, oxyhemoglobin assay, electron paramagnetic resonance (EPR) spectroscopy, mass spectrometry or chemiluminescence, are used to detect/quantify NO or NO-derived metabolites in (plant) extracts or in the headspace of plants (Zeidler *et al.*, 2004; Mur *et al.*, 2011). However, these probably do not reflect the concentrations inside the intact plant cell.

## Breakthrough methodology

The paper presented by Laura Calvo-Begueria and colleagues describes a method that enables detection of NO *in vivo* (Calvo-Begueria *et al.*, 2018). They investigated the formation of the nitrosyl–leghemoglobin complex (Lb^2+^NO) and the production of NO in legume nodules using EPR spectroscopy and the fluorescent specific dye 4,5-diaminofluorescein diacetate (DAF-2 DA), respectively. The EPR method established by the authors allows the detection of Lb^2+^NO in the infection zone of intact nodules (Box 1). Moreover, their work demonstrates that Lb^2+^NO is generated as an artefact when nodules are not analysed immediately after detachment and hence quantification of Lb^2+^NO in nodule extracts is not valid. This confirms that analysis of such reactive compounds should be done using non-invasive methods or at least immediately after sample collection. Finally, their results indicate that EPR complemented by fluorometric methods does allow reliable conclusions about NO production in plants.

**Box 1. Monitoring NO production *in planta* using EPR spectroscopy**

NO binds to the Fe^2+^ of leghemoglobin and forms an Lb^2+^NO nitrosyl complex (left). Calvo-Begueria *et al.* (2018) have demonstrated that this complex can be detected by EPR spectroscopy in intact soybean nodules, allowing a direct monitoring of NO production. Nodules containing Lb^2+^NO show spectra with a clear diagnostic signal in the range of 320–345 mT (right; see Calvo-Begueria *et al.*, 2018). Here, a numerical addition of the spectrum of intact soybean nodules and the spectra of authentic Lb^2+^NO at variable proportions is shown.

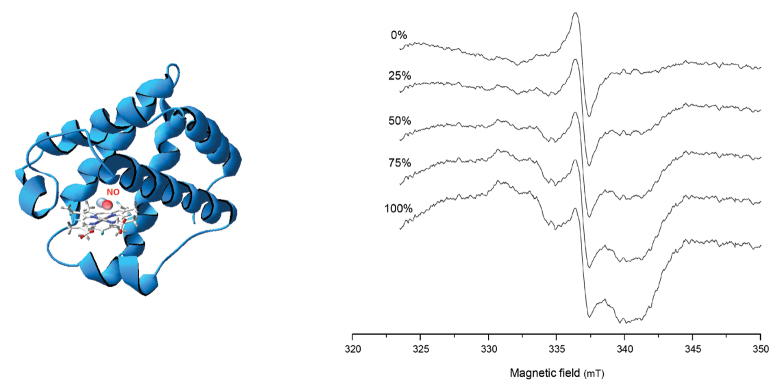

## Perspectives

Although significant progress has been made in developing methods for NO research, future efforts should still concentrate on enhancing the sensitivity and specificity of these methods and focus on *in vivo* detection and quantification of NO. Although the method presented by Calvo-Begueria *et al.* (2018) is restricted to NO detection in the nodule-infected tissue containing leghemoglobin, it is certainly a very promising approach that can be further developed as a general NO sensing technique for analysing NO production in other biological systems. For example, transformation of the leghemoglobin coding sequence into other plant species would enable the use of this protein as an NO sensor and thereby the analysis of NO production/quantification via Lb^2+^NO detection. However, the availability of EPR spectroscopy might be a restriction for using this technology as a standard method in NO research.

In general, an NO sensor protein is an optimal tool for NO detection/quantification *in vivo*. A fluorescence quenching-based NO probe was designed by Eroglu *et al.* (2016). Fusing a bacteria-derived NO-binding domain close to distinct fluorescent protein variants enables a direct observation and quantification of NO. Such genetically encoded NO probes (geNOps) provide a selective, specific and real-time read-out of cellular NO dynamics and, hence, open a new era for NO bio-imaging. Furnished with compartment-specific signal peptides, high-resolution, intracellular NO detection would be possible.

Despite an increasing number of reports on the biological action of NO in plants, the validity of such work should be questioned depending on the manner in which NO has been measured and/or the solution composition used for NO quantification. Therefore, a re-evaluation of past findings is probably needed in some cases. The different measurement techniques that can be used for a given sample type are highlighted in Box 2. Ideally, methods for determination/quantification of NO should exhibit a high degree of sensitivity and specificity, and should in particular facilitate the detection of NO *in planta*. NO sensor proteins (Lb^2+^NO and geNOps) have the potential to fulfil all these ideal characteristics and could revolutionize the field of NO research in plants. Further development of such NO measurement approaches, including the use of appropriate signal peptides and spatiotemporal-specific promotor elements, will allow an accurate determination of NO production in different plant systems, tissues and cells, and help to reveal exactly how, when and where NO is produced. Such a method would provide robust results and assuage the controversial discussions on the detection of NO in plants.

**Box 2. Methods used for NO detection in headspace, *in planta* or in plant extracts**

The different methods available to detect and quantify NO are based on its particular physical and chemical properties. The method of choice depends on the biological question that needs to be answered. Some assays detect NO gas emitted from cells, whereas others allow measurement of NO and its derivatives (e.g. N_2_O_3_, NO_2_^–^) in liquid solutions. NO-sensitive dyes, electrodes and sensor proteins allow detection and quantification of NO *in planta*. NO sensor proteins (Lb^2+^NO) under the control of tissue- or cell-specific promotors are especially promising specific and sensitive tools for spatiotemporal detection of NO *in planta*. However, the use of NO sensor proteins requires suitable detection instruments, such as an EPR spectrometer, a confocal laser scanning microscope, or a chemiluminescence detector. The high cost of these pieces of equipment and the considerable expertise needed to work with them may limit their use for standard methods in NO research.